# Air Pollution, Residential Greenness and Metabolic Dysfunction during Early Pregnancy in the INfancia y Medio Ambiente (INMA) Cohort

**DOI:** 10.3390/ijerph18179354

**Published:** 2021-09-04

**Authors:** Amal Rammah, Kristina W. Whitworth, Christopher I. Amos, Marisa Estarlich, Mònica Guxens, Jesús Ibarluzea, Carmen Iñiguez, Mikel Subiza-Pérez, Martine Vrijheid, Elaine Symanski

**Affiliations:** 1 Center for Precision Environmental Health, Baylor College of Medicine, Houston, TX 77030, USA; amal.rammah@bcm.edu (A.R.); kristina.whitworth@bcm.edu (K.W.W.); 2 Department of Medicine, Section of Epidemiology and Population Sciences, Baylor College of Medicine, Houston, TX 77030, USA; chris.amos@bcm.edu; 3 Institute of Clinical and Translational Medicine, Baylor College of Medicine, Houston, TX 77030, USA; 4 Spanish Consortium for Research on Epidemiology and Public Health (CIBERESP), Instituto de Salud Carlos III, 28029 Madrid, Spain; M.Luisa.Estarlich@uv.es (M.E.); monica.guxens@isglobal.org (M.G.); mambien3-san@euskadi.eus (J.I.); carmen.iniguez@uv.es (C.I.); mikel.subiza@ehu.eus (M.S.-P.); martine.vrijheid@isglobal.org (M.V.); 5 Department of Nursing, University of Valencia, 46010 Valencia, Spain; 6 Epidemiology and Environmental Health Joint Research Unit, The Foundation for the Promotion of Health and Biomedical Research of Valencia Region, Universitat Jaume I-Universitat de València, 46010 Valencia, Spain; 7 ISGlobal, 08003 Barcelona, Spain; 8 Department of Experimental and Health Sciences, Pompeu Fabra University, 08003 Barcelona, Spain; 9 Department of Child and Adolescent Psychiatry, Erasmus University Medical Center (Erasmus MC), 3015 Rotterdam, The Netherlands; 10 Group of Environmental Epidemiology and Child Development, Biodonostia Health Research Institute, 20014 San Sebastian, Spain; 11 Faculty of Psychology, University of the Basque Country UPV/EHU, 20018 San Sebastian, Spain; 12 Ministry of Health of the Basque Government, Sub Directorate for Public Health and Addictions of Gipuzkoa, 20013 San Sebastián, Spain; 13 Department of Statistics and Operational Research, University of Valencia, 46010 Valencia, Spain; 14 Department of Clinical and Health Psychology and Research Methods, University of the Basque Country UPV/EHU, 20018 San Sebastián, Spain

**Keywords:** PM_2.5_, NO_2_, residential greenness, gestational diabetes, GDM, lipids

## Abstract

Despite extensive study, the role of air pollution in gestational diabetes remains unclear, and there is limited evidence of the beneficial impact of residential greenness on metabolic dysfunction during pregnancy. We used data from mothers in the Spanish INfancia y Medio Ambiente (INMA) Project from 2003–2008. We obtained spatiotemporally resolved estimates of fine particulate matter (PM_2.5_) and nitrogen dioxide (NO_2_) exposures in early pregnancy and estimated residential greenness using satellite-based Normal Difference Vegetation Index (NDVI) within 100, 300 and 500 m buffers surrounding the mother’s residence. We applied logistic regression models to evaluate associations between each of the three exposures of interest and (a) glucose intolerance and (b) abnormal lipid levels. We found limited evidence of associations between increases in PM_2.5_ and NO_2_ exposures and the metabolic outcomes. Though not statistically significant, high PM_2.5_ exposure (≥25 µg/m^3^) was associated with increased odds of glucose intolerance (OR = 1.16, 95% CI: 0.82, 1.63) and high cholesterol (OR = 1.14, 95% CI: 0.90, 1.44). High NO_2_ exposure (≥39.8 µg/m^3^) was inversely associated with odds of high triglycerides (OR = 0.70, 95% CI: 0.45, 1.08). Whereas NDVI was not associated with glucose intolerance, odds of high triglycerides were increased, although the results were highly imprecise. Results were unchanged when the air pollutant variables were included in the regression models. Given the equivocal findings in our study, additional investigations are needed to assess effects of air pollution and residential greenness on metabolic dysfunction during pregnancy.

## 1. Introduction

Metabolic disorders of pregnancy, such as impaired glucose intolerance (IGT) or gestational diabetes mellitus (GDM), are associated with an increased risk of cardiovascular diseases [1] and type 2 diabetes post pregnancy [2]. Women with pregnancies complicated by glucose intolerance also have abnormal serum lipid levels, which are markers of metabolic dysfunction [3] and increase mothers’ risks for cardiovascular disease [4]. Metabolic dysfunction in early pregnancy is also associated with increased risk of several adverse birth outcomes [5,6,7,8] and places the infant at risk for cardiometabolic consequences later in life [9].

Exposure to ambient air pollution, including fine particulate matter (PM_2.5_) and nitrogen dioxide (NO_2_), has been linked to oxidative stress and systemic inflammation [10,11,12], and inflammatory mediators are associated with levels of glucose during pregnancy [13]. Air pollution is also associated with lipid oxidization and altered lipid metabolism in animal models [14]. Furthermore, some constituents of particulate matter are endocrine disruptors, which may have potentially adverse effects on pregnancy, including metabolic diseases [15]. While the literature supports an association between air pollution and type 2 diabetes [16], six recent meta-analyses reported mixed results for air pollution and GDM [17,18,19,20,21,22]. More recently, investigations have applied land use regression (LUR) models based on data from monitoring networks [23,24,25,26,27]. Others used a mixture of satellite simulation and monitoring data to predict spatially- and temporally-resolved estimates, although these either assigned exposures at the census mesh block level [28] or the delivery hospital [29] rather than the mother’s residential address, or did not assess residential mobility during pregnancy [30]. Further, to our knowledge, no studies have explored the impact of air pollution exposure on subclinical metabolic disturbances during pregnancy, such as serum lipids. 

While air pollution has detrimental effects on health and well-being, there is growing recognition of the benefits of green space [31]. Investigations have reported associations between residing in areas with higher greenness and lower risk of type II diabetes [32] and lower blood lipid levels [33,34,35]. Further, residential proximity to urban vegetative cover and tree canopy has been linked to decreased allostatic load and improved metabolic function [36]. Also relevant to pregnancy health, there is evidence suggesting a beneficial impact of residential greenness on reducing risks for birthweight and small-for-gestational age (SGA) [37]. Yet only four studies [38,39,40,41] have investigated—with mixed findings—the impact of residential greenness on maternal blood glucose levels, impaired glucose tolerance, and/or gestational diabetes mellitus. Like air pollution, no investigations have examined the impact of greenness on serum lipid levels. Thus, our objective was to examine associations of air pollution and residential greenness with glucose intolerance and abnormal serum lipids among pregnant women. 

## 2. Materials and Methods

### 2.1. Study Population

We used data from the INfancia y Medio Ambiente (INMA) Project, a population-based birth cohort study that recruited mother-infant pairs from multiple regions in Spain with the goal of studying the impact of the environment on pregnancy and child health outcomes [42]. Between 2003 and 2008, eligible women (≥16 years of age; singleton pregnancy; no assisted reproduction; intention to deliver at the recruitment site; and no communication issues) were recruited at around 10–13 weeks’ gestation and followed through delivery. In addition to physical examinations and collection of biological samples, a wide array of maternal sociodemographic, health and lifestyle characteristics were collected via electronic medical record abstraction and questionnaires administered by trained research staff during the first and third trimester. In this study, data were available on 2270 mothers from Gipuzkoa (n = 638), Sabadell (n = 777) and Valencia (n = 855); 2263 remained for analysis after the exclusion of seven pregnant women with diabetes at recruitment. The INMA study was approved by the ethics committee at all reference hospitals from which women were recruited and all women provided written informed consent prior to enrollment. The current study was approved by the Institutional Review Board at Baylor College of Medicine.

### 2.2. Glucose Intolerance and Lipid Levels

Impaired glucose tolerance (IGT) and gestational diabetes mellitus (GDM) were abstracted from electronic medical records. Between the 24th–28th weeks’ gestation, women who were at high risk for IGT or GDM were administered a 50 g oral glucose challenge test (OGCT); an individual with a blood glucose level ≥140 mg/dL one hour after the test was administered a 3 h, 100 g oral glucose tolerance test (OGTT). Women were diagnosed with GDM when their blood glucose levels were at baseline and 1, 2 and 3 h post-OGTT exceeded reference values of the National Diabetes Data Group (NDDG) [43]. IGT was diagnosed when one or fewer of those four glucose concentrations exceed reference values. IGT was not available for women in the Valencia cohort. For analysis in this study, we classified women with IGT and/or GDM as having glucose intolerance and women who had neither IGT nor GDM as not having glucose intolerance. Total serum cholesterol and triglycerides (mg/dL) were measured in non-fasting blood samples obtained at 12 weeks’ gestation. We dichotomized serum lipids as high vs. normal using the non-fasting clinical cutoff values of 190 mg/dL and 175 mg/dL for total cholesterol and total triglycerides, respectively [44].

### 2.3. NO_2_ and PM_2.5_ Exposures

We used LUR spatial estimates from models previously developed as part of the ESCAPE project [45] to obtain exposure estimates of NO_2_ at each geocoded residential address. We then applied back-extrapolation procedures similar to those in ESCAPE [46] to temporally adjust these estimates using the ratio of NO_2_ concentrations measured by background monitors in each study area (i.e., Sabadell, Gipuzkoa, or Valencia) on each day of the study period (i.e., between 2003 and 2008) to the annual average in 2009. Because data on residential mobility were known, NO_2_ exposures were estimated based on the residence at which women lived on that day. For this study, daily NO_2_ exposures were averaged for trimesters one and two.

Similar to methods previously described [47], we used satellite-derived Multi-Angle Implementation of Atmospheric Correction (MAIAC) algorithm aerosol optical depth (AOD) measurements coupled with land-use predictors and meteorological parameters, to produce spatially resolved daily PM_2.5_ exposure estimates at 1 km resolution across Spain for 2009 to 2016. Due to limited ambient PM_2.5_ monitoring stations in our study areas prior to 2009, we temporally-adjusted the 2009 PM_2.5_ estimates using daily time-series data from local background monitoring stations for PM_2.5_ (in Gipuzkoa) and PM_10_ (in Sabadell and Valencia), similar to the methods used in ESCAPE [46]. We used as an adjustment factor the ratio of measured pollution concentrations from the background monitor on the day for which we wanted to estimate exposure to measurements on the same day in 2009. As with the NO_2_ exposure estimates, PM_2.5_ exposures were estimated for each residential address women reported and daily PM_2.5_ estimates were averaged for trimesters one and two.

### 2.4. Residential Greenness

**Figure 1:**
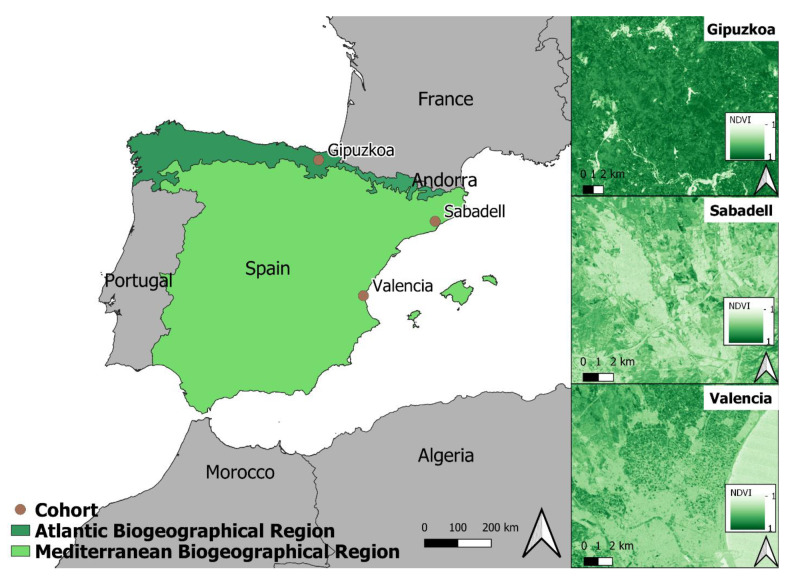
NDVI maps and biogeographic regions of the three INMA sub-cohorts, Gipuzkoa, Sabadell and Valencia, 2003–2008.

We used the Normalized Difference Vegetation Index (NDVI) as a measure of residential greenness. NDVI is based on the difference in the amount of visible (red) and near-infrared parts of the spectrum that are reflected by land surfaces [48]. NDVI values range from −1 to 1, and higher numbers indicate more greenness. To achieve maximum exposure contrast, we used available cloud-free Landsat images (Landsat 4–5 TM data at 30 m × 30 m resolution) on days during the greenest months for each cohort when clear-sky (cloud-free) satellite data were available: in Spring for Gipuzkoa (in the Atlantic biogeographic region) and in Winter for Sabadell and Valencia (in the Mediterranean biogeographic region) (Figure 1). Average NDVI was estimated in buffers of 100, 300 and 500 m surrounding a mother’s residential address during the first and second trimesters, considering any changes in residence during that period. We also obtained information on the distance from residence to the nearest large green space (≥5000 m squared (m^2^)), as well as the availability of large green spaces within a 300 m distance from the residence.

### 2.5. Covariates

The following sociodemographic and neighborhood covariates were evaluated as potential confounders: education (primary, secondary, university), social class (based on occupation: managers and professionals, technicians and associate professionals; other skilled labor; skilled, semi-skilled or unskilled manual labor), urbanicity of residence during the first trimester (urban vs. rural) and a self-reported measure of noise disturbance. Relevant pregnancy and lifestyle characteristics that were evaluated as confounders included age at last menstrual period, parity, gravidity, gestational weight gain (within, above and below Institute of Medicine Guidelines [49]), physical activity (in metabolic equivalents of task (METs) per hour per day) in the last year and during the first trimester, smoking and alcohol consumption in the first trimester and body mass index (BMI) (kilograms (kg)/meters squared (m^2^)) based on self-reported pre-pregnancy weight and height. We also obtained information on diet via a validated food frequency questionnaire administered in the first trimester and computed a proxy measure of a healthy diet: the relative Mediterranean diet score (rMED) [50].

### 2.6. Statistical Analysis

We applied logistic regression models to separately estimate odds ratios (OR) and 95% confidence intervals for associations between each exposure and each metabolic outcome (i.e., glucose intolerance, total cholesterol, total triglycerides). We evaluated PM_2.5_ and NO_2_ as continuous as well as dichotomized (high vs. low) variables at the 75th percentile (≥25 µg/m^3^ for PM_2.5_; ≥39.8 µg/m^3^ for NO_2_). Results for NDVI and distance to the nearest large green space are presented per interquartile range (IQR) and availability of a large green space within an assigned buffer was evaluated as a dichotomous variable (yes/no). In addition to cohort, we evaluated the covariates listed above as confounders in each univariable model using the10%-change-in-estimate rule [51]. Based on this assessment, all models were adjusted for cohort. Both the dichotomous PM_2.5_- and NO_2_-glucose intolerance models were further adjusted for urbanicity; the NO_2_-glucose intolerance model additionally included noise disturbance. We observed little temporal variability in estimated PM_2.5_, NO_2_ and greenness exposures between the first and second trimester and effect estimates were similar between trimesters (data not shown). NDVI results were also similar irrespective of buffer size. Thus, we present results for first trimester PM_2.5_, NO_2_, 300 m NDVI and other greenness metrics below. We ran additional analyses for residential greenness and each of the metabolic outcomes and examined whether adding PM_2.5_, NO_2_ or both pollutants in the model changed the effect estimates. All analyses were conducted using SAS (version 9.4, SAS Institute Inc., Cary, NC, USA).

## 3. Results

Selected maternal sociodemographic and lifestyle characteristics are presented in Table 1. The mean (SD) age of participants in our study was 30.4 (4.4) years and the majority were born in Spain (88%), married (93%) and had completed either a university (31%) or secondary school education (12 years of schooling) (39%). Most participants had a normal BMI (kg/m^2^) (68%); 25% were overweight or obese. Approximately 63% did not smoke during the first trimester and almost 52% of participants had a score that reflected medium to high adherence to a Mediterranean diet in the first trimester based on the food frequency questionnaire. Also shown in Table 1 is information on the exposure metrics for PM_2.5_, NO_2_ and NDVI for different size buffers. Mean (SD) PM_2.5_ exposure was 28.2 (16) µg/m^3^, which exceeds the WHO air quality guideline of 10 µg/m^3^ annual mean [52]. Table 2 reports on the prevalence of glucose intolerance (IGT/GDM) and elevated serum lipids among INMA women.

**Figure 2:**
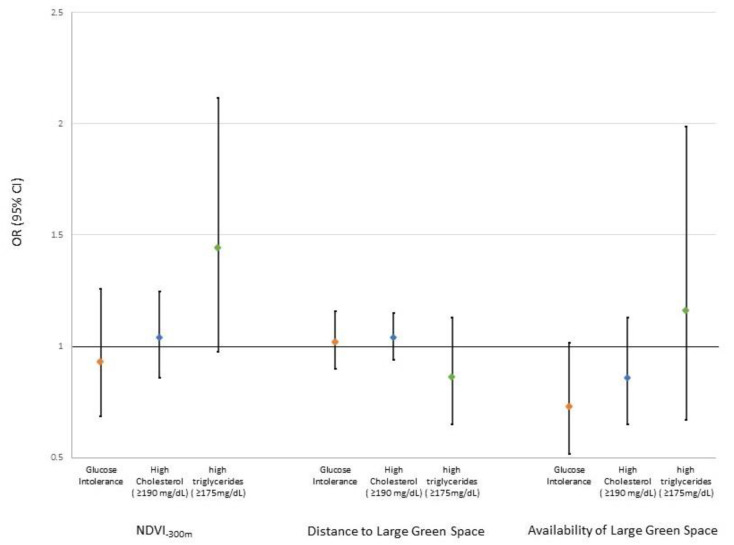
Adjusted odds ratios (OR) and 95% confidence intervals (CI) for the association between increases in residential greenness at 300 m (IQR = 0.19) and distance to (IQR = 162 m) and availability of large green spaces (≥5000 m^2^) within 300 m of residence in the first trimester and glucose intolerance (IGT and/or GDM) and elevated serum lipids among women of the INMA Cohort, 2003–2008.

We observed no statistically significant associations between air pollution exposure and increased odds of either glucose intolerance or elevated cholesterol or triglycerides. However, women who were exposed to high levels of PM_2.5_ had increased odds of glucose intolerance (OR = 1.16, 95% CI: 0.82, 1.63) and high total cholesterol (OR = 1.14, 95% CI: 0.90, 1.44) (Table 3). For associations between triglycerides and NO_2_, unexpectedly, we observed an OR of 0.85 (95% CI: 0.74, 0.98) per 10 μg/m^3^ increase in exposure and an OR of 0.70 (95% CI: 0.45, 1.08) among women in the highest quartile relative to the lowest quartile of exposure. As shown in Figure 2, although not statistically significant, equivocal results for greenness were observed. A 0.19 IQR increase in 300 m NDVI was associated with increased odds of high triglycerides (OR = 1.44, 95% CI: 0.98, 2.12); ORs were closer to the null value for glucose intolerance and high total cholesterol. Availability of a large green space within a 300 m buffer was associated with a reduction in odds for both glucose intolerance (OR = 0.73, 95% CI: 0.52, 1.02) and high cholesterol (OR = 0.86, 95% CI: 0.65, 1.13), whereas we observed increased odds for high triglycerides (OR = 1.16, 95% CI: 0.67, 1.99). To evaluate potential confounding due to air pollution, we ran models that included NO_2_ and PM_2.5_. In all cases, there was little change in the ORs for residential greenness. For example, in models that included both air pollutants, the OR for a 0.19 IQR increase in 300 m NDVI was 1.33 (95% CI: 0.89, 2.01) for high triglycerides (data not shown). Given the lack of statistical significance in our results, we chose not to examine either of these two pollutants as potential mediators of the association between greenness and metabolic dysfunction.

## 4. Discussion

We found equivocal, sometimes conflicting, evidence of associations between air pollution and glucose intolerance and non-fasting serum lipids among women during their pregnancy. While associations were not statistically significant, the odds of glucose intolerance and total cholesterol were increased with exposure to PM_2.5_ or NO_2_. In contrast, we found inverse associations for elevated total triglycerides. Recent meta-analyses on NO_2_ and GDM report either null associations [19,22] or marginal (and imprecise) pooled effect estimates [18,20,21]. Prior evidence on associations between PM_2.5_ and gestational diabetes during pregnancy is also inconsistent [17,18,19,20,21,22]. We also observed differing results for residential greenness depending on which metabolic outcome was under investigation, as well as on which metric was used. For NDVI, results were similar irrespective of the size of the buffer surrounding a mother’s residence. 

Altered lipid metabolism may be influenced by systemic responses to air pollution exposure, such as inflammation and oxidative stress [53], and exposure to fine particulate matter may also play a role in the development of cardiometabolic risk factors through DNA methylation in genes involved in lipid metabolism [54]. While there are physiological changes unique to pregnancy, there are no reference ranges for cholesterol or triglyceride levels for pregnant women [55] and no published studies in similar populations to which we could directly compare our results. Our finding of decreased odds of elevated total triglycerides among women exposed to high levels of NO_2_ is difficult to explain and may be a spurious association. However, results from previous studies of adults generally reported positive associations. For example, a meta-analysis of three studies reported statistically significant increases in triglycerides associated with long-term exposure to PM_10_ and NO_2_, as well as slight increases in total cholesterol [56]. Other investigations reported increases in PM_2.5_ and elevated triglyceride and cholesterol levels in a cross-sectional study of adults in North Carolina [57], and between long-term exposure to PM_2.5_ and NO_2_ and increases in cholesterol and triglycerides among adults living in China [58]. Another study in China found a positive association for NO_2_ with hypertriglyceridemia, but not for PM_2.5_ [59].

Residential greenness may have beneficial physiological and psychological impacts on metabolic health during pregnancy, through the reduction of harmful exposures to air pollution and noise, promoting physical activity and reducing adiposity and stress. Such benefits may differ based on the type, quality and accessibility of nearby green spaces that may be utilized during pregnancy [60]. This may explain the conflicting results (point estimates of ORs ranging from 0.73 to 1.02, all with wide confidence intervals) based on the metrics used for assessing greenness in this study. For GDM and similar to our findings, Young et al. [38] found no association with green space (measured as km^2^ per zip code) in an ecological study in two southern California counties in the U.S.A. A population-based cohort study of women in Rhode Island also found no association between NDVI and GDM [39]. In contrast, two investigations observed protective effects for increases in NDVI exposure on GDM in a case-control study in Guangdong, China [41] and on IGT and GDM in a cohort study in Wuhan, China [40]. A possible explanation for these inconsistencies may be the limited degree of variability in NDVI in the current study (300 m NDVI IQR = 0.19) compared to that in the other two studies (250 m NDVI IQR = 0.28 [41] and 300 m NDVI IQR = 0.27 [40]).

We observed positive, albeit imprecise, associations between greenspace and elevated total triglycerides. While no previous findings of the association between residential greenness and serum lipids among pregnant women are available for comparison, one study evaluating NDVI in public open spaces in Adelaide, Australia also found a positive association (marginally significant) with incident dyslipidemia [61]. In contrast and in the expected direction, other studies reported negative relationships between NDVI and triglyceride levels in a longitudinal study of older adults in London [34], NDVI and total cholesterol and triglyceride levels in a cross-sectional study of Chinese adults [35], and NDVI (measured at the census block rather than within defined buffers surrounding a residence) and hyperlipidemia with greenness among older adults in Florida [33]. 

Our study has several strengths and limitations. While we were able to evaluate serum lipids as subclinical markers of metabolic disturbance early in pregnancy, it was not possible to evaluate a milder form of glucose intolerance, as information on IGT was missing from one cohort. For classifying women as having elevated cholesterol or triglyceride levels, we used clinical cut-off points for non-fasting lipids measurements. Despite their slow uptake into clinical practice, the use of non-fasting lipid measurements is recommended by several clinical guidelines based on evidence of their adequacy in assessing dyslipidemia [62]. One strength of our study was that the air pollution exposure assessment relied on a mixture of satellite-derived data, land use regression modelling and back-extrapolation using fixed monitoring network data. We also evaluated different characteristics of green space and used a validated satellite-based method to estimate residential greenness [63]. We further addressed limitations in previous investigations by considering residential mobility during pregnancy in assessing air pollution exposure and greenness. However, it is not clear at what distance greenness has an impact on metabolic outcomes. Evidence from a systematic review suggests that greenness measured at larger buffer sizes (e.g., ≥500 m) around a residential address may be a better predictor of health outcomes [64], though we did not find this to be the case in our study. We also did not have information on the type of vegetation available within different buffer sizes. Finally, the density of greenspace measured using NDVI may not correspond to the availability of accessible greenspaces, and we did not have information on utilization of greenspaces, such as exercise or recreation in nearby parks.

## 5. Conclusions

Albeit not statistically significant, our study provides suggestive evidence of an association between PM_2.5_ and NO_2_ exposure and glucose intolerance and elevated cholesterol during pregnancy among women of the INMA Cohort. Our mixed findings for residential greenness, which differed by outcome and measure of greenness, do not support the hypothesis of a beneficial impact on health during pregnancy, and future studies should evaluate access and utilization of surrounding greenness during pregnancy to better understand its health benefits. 

## Figures and Tables

**Table 1:** Selected demographic and lifestyle characteristics and exposures during pregnancy, INMA Cohort, 2003–2008.

Characteristic	All Cohorts	Gipuzkoa	Sabadell	Valencia
	N (%)	N (%)	N (%)	N (%)
Age				
Mean ± SD	30.4 ± 4.4	31.4 ± 3.7	30.2 ± 4.6	29.8 ± 4.7
Min–Max	15–45.5	18–43	16.3–45.5	15–43
Missing	16 (0.71)	-	16 (2.1)	-
Education				
Primary school	572 (25.3)	86 (13.5)	201 (25.9)	28 (3.3)
Secondary school	889 (39.3)	232 (36.5)	308 (39.6)	285 (35.5)
University	708 (31.3)	316 (49.7)	204 (26.3)	349 (41.1)
Missing	94 (4.2)	2 (0.3)	64 (8.2)	188 (22.1)
Total Physical Activity (METs/hour/day) in previous year, first trimester				
Mean ± SD	37.9 ± 3.7	38.2 ± 3.6	37.9 ± 3.7	37.7 ± 3.8
Min–Max	29–54	31.2–51.6	30.2–54	29–53
Missing	168 7.4)	13 (2)	123 (15.8)	32 (3.8)
Relative Mediterranean diet score (rMED), first trimester				
Low score (1–7)	925 (40.9)	167 (26.3)	289 (37.2)	469 (55.2)
Medium score (8–9)	579 (25.6)	192 (30.2)	190 (24.5)	197 (23.2)
High score (10–15)	593 (26.2)	267 (42)	175 (22.5)	151 (17.8)
Missing	166 (7.3)	10 (1.6)	123 (15.8)	33 (3.9)
Body Mass Index (BMI)				
Under weight (<18.5 kg/m^2^)	103 (4.6)	24 (3.8)	37 (4.8)	42 (4.9)
Normal weight (≥18.5 and <25 kg/m^2^)	1531 (67.7)	480 (75.5)	500 (64.4)	551 (64.8)
Overweight (≥25 & <30 kg/m^2^)	395 (17.5)	101 (15.9)	145 (18.7)	149 (17.5)
Obese (≥30 kg/m^2^)	177 (7.8)	31 (4.9)	68 (8.8)	78 (9.2)
Missing	57 (2.5)	-	27 (3.5)	30 (3.5)
Smoking, first trimester				
No	1429 (63.2)	519 (81.6)	601 (77.4)	587 (69.1)
Yes	675 (29.8)	77 (12.1)	119 (15.3)	195 (22.9)
Missing	159 (7.0)	40 (6.3)	57 (7.3)	68 (8)
Alcohol Consumption, first trimester				
Mean ± SD	0.3 ± 1.3	0.2 ± 0.7	0.4 ± 1.4	0.4 ± 1.5
Min–Max	0–15.2	0–7.8	0–15.2	0–14.4
Missing	166 (7.3)	10 (1.6)	123 (15.8)	33 (3.9)
Gravidity				
1	992 (43.8)	282 (44.3)	333 (42.9)	377 (44.4)
2	788 (34.8)	245 (38.5)	271 (34.9)	272 (32)
3+	443 (19.6)	109 (17.1)	161 (20.7)	173 (20.4)
Missing	40 (1.8)	-	12 (1.5)	28 (3.3)
Gestational Weight Gain				
Within IOM Guidelines	745 (32.9)	227 (35.7)	243 (31.3)	275 (32.4)
Below IOM Guidelines	468 (20.7)	194 (30.5)	116 (14.9)	158 (18.6)
Above IOM Guidelines	720 (31.8)	140 (22.0)	240 (30.9)	340 (40)
Missing	330 (14.6)	75 (11.8)	178 (22.9)	77 (9.06)
Urbanicity of residence				
Semi-urban/rural	122 (5.4)	316 (49.7)	-	121 (14.2)
Urban	1704 (75.3)	320 (50.3)	656 (84.4)	728 (85.7)
Missing	122 (5.4)	-	121 (15.6)	1 (0.12)
PM_2.5_ (µg/m^3^) Mean ± SD	21.3 ± 5.2	16.2 ± 2.1	21.9 ± 4.0	24.6 ± 4.8
NO_2_ (µg/m^3^) Mean ± SD	28.2 ± 16.0	14.4 ± 4.3	37.7 ± 13.1	31.1 ± 16.8
NDVI 100 m Mean ± SD	0.2 ± 0.1	0.4 ± 0.1	0.2 ± 0.1	0.2 ± 0.1
NDVI 300 m Mean ± SD	0.3 ± 0.1	0.4 ± 0.1	0.2 ± 0.1	0.2 ± 0.1
NDVI 500 m Mean ± SD	0.3 ± 0.2	0.5 ± 0.1	0.2 ± 0.1	0.2 ± 0.1

MET: metabolic equivalents of task; IOM: Institute of Medicine; NDVI: Normalized Difference Vegetation Index.

**Table 2:** Prevalence of glucose intolerance (IGT/GDM) and elevated serum lipids among women of the INMA Cohort, 2003–2008.

Outcome	All Cohorts	Gipuzkoa	Sabadell	Valencia
	N (%)	N (%)	N (%)	N (%)
Glucose intolerance				
No	1682 (74.3)	538 (84.6)	390 (50.2)	754 (88.7)
Yes	268 (11.8)	40 (6.3)	186 (23.9)	42 (4.9)
Missing	313 (13.8)	58 (9.1)	201 (25.9)	54 (6.4)
Total Cholesterol				
Normal (<190 mg/dL)	915 (40.4)	299 (47.0)	332 (42.7)	284 (33.4)
High (≥190 mg/dL)	1074 (47.5)	321 (50.5)	302 (38.9)	451 (53.1)
Missing	274 (12.1)	16 (2.5)	143 (18.4)	115 (13.5)
Total Triglycerides				
Normal (<175 mg/dL)	1857 (82.1)	598 (94.0)	585 (75.3)	674 (79.3)
High (≥175 mg/dL)	130 (5.7)	22 (3.5)	49 (6.3)	59 (6.9)
Missing	276 (12.2)	16 (2.5)	143 (18.4)	117 (13.8)

**Table 3:** Adjusted odds ratios (OR) ^a^ and 95% confidence intervals (CI) for the association between PM_2.5_ and NO_2_ exposure in the first trimester and glucose intolerance (IGT and/or GDM) and elevated serum lipids among women of the INMA Cohort, 2003–2008.

Outcome	Glucose Intolerance	High Total Cholesterol (≥190 mg/dL)	High Total Triglycerides (≥175 mg/dL)
	OR (95% CI)	OR (95% CI)	OR (95% CI)
PM_2.5_			
per 5 µg/m^3^	1.02 (0.85, 1.21)	0.99 (0.88, 1.11)	0.95 (0.76, 1.18)
High (≥25 µg/m^3^)	1.16 (0.82 1.63) ^b^	1.14 (0.90, 1.44)	0.89 (0.58, 1.37)
NO_2_			
per 10 µg/m^3^	0.99 (0.89, 1.11)	1.05 (0.98, 1.13)	0.85 (0.74, 0.98)
High (≥39.8 µg/m^3^)	1.05 (0.76, 1.44) ^c^	1.11 (0.88, 1.39)	0.70 (0.45, 1.08)

^a^ All models adjusted for cohort. ^b^ Additionally adjusted for urbanicity of residence in the first trimester. ^c^ Additionally adjusted for urbanicity of residence in the first trimester and noise disturbance.

## Data Availability

Data are available upon reasonable request by contacting inma@proyectoinma.org. Information regarding the INMA Collaboration Policy is available here: https://www.proyectoinma.org/en/inma-project/inma-collaboration-policy/ (accessed on 2 September 2021).

## References

[1] Kramer C.K., Campbell S., Retnakaran R. (2019). Gestational diabetes and the risk of cardiovascular disease in women: A systematic review and meta-analysis. Diabetologia.

[2] Dennison R.A., Chen E.S., Green M.E., Legard C., Kotecha D., Farmer G., Sharp S.J., Ward R.J., Usher-Smith J.A., Griffin S.J. (2020). The absolute and relative risk of type 2 diabetes after gestational diabetes: A systematic review and meta-analysis of 129 studies. Diabetes Res. Clin. Pract..

[3] Ryckman K.K., Spracklen C.N., Smith C.J., Robinson J.G., Saftlas A.F. (2015). Maternal lipid levels during pregnancy and gestational diabetes: A systematic review and meta-analysis. BJOG.

[4] Wild R., Weedin E.A., Wilson D. (2015). Dyslipidemia in pregnancy. Cardiol. Clin..

[5] Enquobahrie D.A., Williams M.A., Butler C.L., Frederick I.O., Miller R.S., Luthy D.A. (2004). Maternal plasma lipid concentrations in early pregnancy and risk of preeclampsia. Am. J. Hypertens..

[6] Vrijkotte T.G., Krukziener N., Hutten B.A., Vollebregt K.C., van Eijsden M., Twickler M.B. (2012). Maternal lipid profile during early pregnancy and pregnancy complications and outcomes: The ABCD study. J. Clin. Endocrinol. Metab..

[7] Farrar D., Simmonds M., Bryant M., Sheldon T.A., Tuffnell D., Golder S., Dunne F., Lawlor D.A. (2016). Hyperglycaemia and risk of adverse perinatal outcomes: Systematic review and meta-analysis. BMJ.

[8] Wang J., Moore D., Subramanian A., Cheng K.K., Toulis K.A., Qiu X., Saravanan P., Price M.J., Nirantharakumar K. (2018). Gestational dyslipidaemia and adverse birthweight outcomes: A systematic review and meta-analysis. Obes. Rev..

[9] Nijs H., Benhalima K. (2020). Gestational diabetes mellitus and the long-term risk for glucose intolerance and overweight in the offspring: A narrative review. J. Clin. Med..

[10] Lee P.C., Talbott E.O., Roberts J.M., Catov J.M., Sharma R.K., Ritz B. (2011). Particulate air pollution exposure and C-reactive protein during early pregnancy. Epidemiology.

[11] Yi L., Wei C., Fan W. (2017). Fine-particulate matter (PM2.5), a risk factor for rat gestational diabetes with altered blood glucose and pancreatic GLUT2 expression. Gynecol. Endocrinol..

[12] Lim C.C., Thurston G.D. (2019). Air pollution, oxidative stress, and diabetes: A life course epidemiologic perspective. Curr. Diabetes Rep..

[13] Lowe L.P., Metzger B.E., Lowe W.L., Dyer A.R., McDade T.W., McIntyre H.D., HAPO Study Cooperative Research Group (2010). Inflammatory mediators and glucose in pregnancy: Results from a subset of the hyperglycemia and adverse pregnancy Outcome (HAPO) Study. J. Clin. Endocrinol. Metab..

[14] Li R., Navab M., Pakbin P., Ning Z., Navab K., Hough G., Morgan T.E., Finch C.E., Araujo J.A., Fogelman A.M. (2013). Ambient ultrafine particles alter lipid metabolism and HDL anti-oxidant capacity in LDLR-null mice. J. Lipid Res..

[15] Darbre P.D. (2018). Overview of air pollution and endocrine disorders. Int. J. Gen. Med..

[16] Eze I.C., Hemkens L.G., Bucher H.C., Hoffmann B., Schindler C., Kunzli N., Schikowski T., Probst-Hensch N.M. (2015). Association between ambient air pollution and diabetes mellitus in Europe and North America: Systematic review and meta-analysis. Environ. Health Perspect..

[17] He D., Wu S., Zhao H., Qiu H., Fu Y., Li X., He Y. (2017). Association between particulate matter 2.5 and diabetes mellitus: A meta-analysis of cohort studies. J. Diabetes Investig..

[18] Elshahidi M.H. (2019). Outdoor air pollution and gestational diabetes mellitus: A systematic review and meta-analysis. Iran. J. Public Health.

[19] Bai W., Li Y., Niu Y., Ding Y., Yu X., Zhu B., Duan R., Duan H., Kou C., Li Y. (2020). Association between ambient air pollution and pregnancy complications: A systematic review and meta-analysis of cohort studies. Environ. Res..

[20] Hu C.Y., Gao X., Fang Y., Jiang W., Huang K., Hua X.G., Yang X.J., Chen H.B., Jiang Z.X., Zhang X.J. (2020). Human epidemiological evidence about the association between air pollution exposure and gestational diabetes mellitus: Systematic review and meta-analysis. Environ. Res..

[21] Tang X., Zhou J.B., Luo F., Han Y., Heianza Y., Cardoso M.A., Qi L. (2020). Air pollution and gestational diabetes mellitus: Evidence from cohort studies. BMJ Open Diabetes Res. Care.

[22] Zhang H., Wang Q., He S., Wu K., Ren M., Dong H., Di J., Yu Z., Huang C. (2020). Ambient air pollution and gestational diabetes mellitus: A review of evidence from biological mechanisms to population epidemiology. Sci. Total Environ..

[23] Kang J., Liao J., Xu S., Xia W., Li Y., Chen S., Lu B. (2020). Associations of exposure to fine particulate matter during pregnancy with maternal blood glucose levels and gestational diabetes mellitus: Potential effect modification by ABO blood group. Ecotoxicol. Environ. Saf..

[24] Ye B., Zhong C., Li Q., Xu S., Zhang Y., Zhang X., Chen X., Huang L., Wang H., Zhang Z. (2020). The associations of ambient fine particulate matter exposure during pregnancy with blood glucose levels and gestational diabetes mellitus risk: A prospective cohort study in Wuhan, China. Am. J. Epidemiol..

[25] Zhang H., Dong H., Ren M., Liang Q., Shen X., Wang Q., Yu L., Lin H., Luo Q., Chen W. (2020). Ambient air pollution exposure and gestational diabetes mellitus in Guangzhou, China: A prospective cohort study. Sci. Total Environ..

[26] Zhang M., Wang X., Yang X., Dong T., Hu W., Guan Q., Tun H.M., Chen Y., Chen R., Sun Z. (2020). Increased risk of gestational diabetes mellitus in women with higher prepregnancy ambient PM (2.5) exposure. Sci. Total Environ..

[27] Hehua Z., Yang X., Qing C., Shanyan G., Yuhong Z. (2021). Dietary patterns and associations between air pollution and gestational diabetes mellitus. Environ. Int..

[28] Melody S.M., Wills K., Knibbs L.D., Ford J., Venn A., Johnston F. (2020). Maternal exposure to ambient air pollution and pregnancy complications in Victoria, Australia. Int. J. Environ. Res. Public Health.

[29] Yu G., Ao J., Cai J., Luo Z., Martin R., Donkelaar A.V., Kan H., Zhang J. (2020). Fine particular matter and its constituents in air pollution and gestational diabetes mellitus. Environ. Int..

[30] Fleisch A.F., Kloog I., Luttmann-Gibson H., Gold D.R., Oken E., Schwartz J.D. (2016). Air pollution exposure and gestational diabetes mellitus among pregnant women in Massachusetts: A cohort study. Environ. Health.

[31] Fong K.C., Hart J.E., James P. (2018). A review of epidemiologic studies on greenness and health: Updated literature through 2017. Curr. Environ. Health Rep..

[32] den Braver N.R., Lakerveld J., Rutters F., Schoonmade L.J., Brug J., Beulens J.W.J. (2018). Built environmental characteristics and diabetes: A systematic review and meta-analysis. BMC Med..

[33] Brown S.C., Lombard J., Wang K., Byrne M.M., Toro M., Plater-Zyberk E., Feaster D.J., Kardys J., Nardi M.I., Perez-Gomez G. (2016). Neighborhood greenness and chronic health conditions in medicare beneficiaries. Am. J. Prev. Med..

[34] de Keijzer C., Basagaña X., Tonne C., Valentín A., Alonso J., Antó J.M., Nieuwenhuijsen M.J., Kivimäki M., Singh-Manoux A., Sunyer J. (2019). Long-term exposure to greenspace and metabolic syndrome: A whitehall II study. Environ. Pollut..

[35] Yang B.Y., Markevych I., Heinrich J., Bloom M.S., Qian Z., Geiger S.D., Vaughn M., Liu S., Guo Y., Dharmage S.C. (2019). Residential greenness and blood lipids in urban-dwelling adults: The 33 Communities Chinese Health Study. Environ. Pollut..

[36] Egorov A.I., Griffin S.M., Converse R.R., Styles J.N., Sams E.A., Wilson A., Jackson L.E., Wade T.J. (2017). Vegetated land cover near residence is associated with reduced allostatic load and improved biomarkers of neuroendocrine, metabolic and immune functions. Environ. Res..

[37] Zhan Y., Liu J., Lu Z., Yue H., Zhang J., Jiang Y. (2020). Influence of residential greenness on adverse pregnancy outcomes: A systematic review and dose-response meta-analysis. Sci. Total Environ..

[38] Young C., Laurent O., Chung J.H., Wu J. (2016). Geographic distribution of healthy resources and adverse pregnancy outcomes. Matern. Child Health J..

[39] Choe S.A., Kauderer S., Eliot M.N., Glazer K.B., Kingsley S.L., Carlson L., Awad Y.A., Schwartz J.D., Savitz D.A., Wellenius G.A. (2018). Air pollution, land use, and complications of pregnancy. Sci. Total Environ..

[40] Liao J., Chen X., Xu S., Li Y., Zhang B., Cao Z., Zhang Y., Liang S., Hu K., Xia W. (2019). Effect of residential exposure to green space on maternal blood glucose levels, impaired glucose tolerance, and gestational diabetes mellitus. Environ. Res..

[41] Qu Y., Yang B., Lin S., Bloom M.S., Nie Z., Ou Y., Mai J., Wu Y., Gao X., Dong G. (2020). Associations of greenness with gestational diabetes mellitus: The guangdong registry of Congenital Heart Disease (GRCHD) study. Environ. Pollut..

[42] Guxens M., Ballester F., Espada M., Fernandez M.F., Grimalt J.O., Ibarluzea J., Olea N., Rebagliato M., Tardon A., Torrent M. (2012). Cohort profile: The INMA--INfancia y Medio Ambiente--(Environment and childhood) project. Int. J. Epidemiol..

[43] Mellitus D. (2003). Expert Committee on the Diagnosis and Classification of Diabetes Mellitus, Report of the expert committee on the diagnosis and classification of diabetes mellitus. Diabetes Care.

[44] Nordestgaard B.G., Langsted A., Mora S., Kolovou G., Baum H., Bruckert E., Watts G.F., Sypniewska G., Wiklund O., Borén J. (2016). Fasting is not routinely required for determination of a lipid profile: Clinical and laboratory implications including flagging at desirable concentration cut-points-a joint consensus statement from the European atherosclerosis society and european federation of clinical chemistry and laboratory medicine. Eur. Heart J..

[45] Beelen R., Hoek G., Vienneau D., Eeftens M., Dimakopoulou K., Pedeli X., Tsai M.-Y., Künzli N., Schikowski T., Marcon A. (2013). Development of NO2 and NOx land use regression models for estimating air pollution exposure in 36 study areas in Europe–the ESCAPE project. Atmos. Environ..

[46] Pedersen M., Giorgis-Allemand L., Bernard C., Aguilera I., Andersen A.M., Ballester F., Beelen R.M., Chatzi L., Cirach M., Danileviciute A. (2013). Ambient air pollution and low birthweight: A European cohort study (ESCAPE). Lancet Respir. Med..

[47] Stafoggia M., Bellander T., Bucci S., Davoli M., de Hoogh K., De’ Donato F., Gariazzo C., Lyapustin A., Michelozzi P., Renzi M. (2019). Estimation of daily PM (10) and PM (2.5) concentrations in Italy, 2013–2015, using a spatiotemporal land-use random-forest model. Environ. Int..

[48] Weier J., Herring D. Measuring Vegetation (NDVI & EVI). https://earthobservatory.nasa.gov/features/MeasuringVegetation.

[49] Institute of Medicine and National Research Council (2010). Weight Gain during Pregnancy: Reexamining the Guidelines.

[50] Fernández-Barrés S., Romaguera D., Valvi D., Martínez D., Vioque J., Navarrete-Muñoz E.M., Amiano P., Gonzalez-Palacios S., Guxens M., Pereda E. (2016). Mediterranean dietary pattern in pregnant women and offspring risk of overweight and abdominal obesity in early childhood: The INMA birth cohort study. Pediatr. Obes..

[51] Rothman K.J., Greenland S., Lash T.L. (2015). Modern Epidemiology.

[52] (2006). Air Quality Guidelines: Global Update 2005: Particulate Matter, Ozone, Nitrogen Dioxide and Sulfur Dioxide.

[53] Li J., Zhou C., Xu H., Brook R.D., Liu S., Yi T., Wang Y., Feng B., Zhao M., Wang X. (2019). Ambient air pollution is associated with HDL (High-Density Lipoprotein) dysfunction in healthy adults. Arterioscler. Thromb. Vasc. Biol..

[54] Li H., Chen R., Cai J., Cui X., Huang N., Kan H. (2018). Short-term exposure to fine particulate air pollution and genome-wide DNA methylation: A randomized, double-blind, crossover trial. Environ. Int..

[55] Bartels Ä., O’Donoghue K. (2011). Cholesterol in pregnancy: A review of knowns and unknowns. Obstet. Med..

[56] Gaio V., Roquette R., Dias C.M., Nunes B. (2019). Ambient air pollution and lipid profile: Systematic review and meta-analysis. Environ. Pollut..

[57] McGuinn L.A., Schneider A., McGarrah R.W., Ward-Caviness C., Neas L.M., Di Q., Schwartz J., Hauser E.R., Kraus W.E., Cascio W.E. (2019). Association of long-term PM (2.5) exposure with traditional and novel lipid measures related to cardiovascular disease risk. Environ. Int..

[58] Yang B.Y., Bloom M.S., Markevych I., Qian Z.M., Vaughn M.G., Cummings-Vaughn L.A., Li S., Chen G., Bowatte G., Perret J.L. (2018). Exposure to ambient air pollution and blood lipids in adults: The 33 communities Chinese health study. Environ. Int..

[59] Yang B.Y., Guo Y., Markevych I., Qian Z.M., Bloom M.S., Heinrich J., Dharmage S.C., Rolling C.A., Jordan S.S., Komppula M. (2019). Association of long-term exposure to ambient air pollutants with risk factors for cardiovascular disease in China. JAMA Netw. Open.

[60] Markevych I., Schoierer J., Hartig T., Chudnovsky A., Hystad P., Dzhambov A.M., de Vries S., Triguero-Mas M., Brauer M., Nieuwenhuijsen M.J. (2017). Exploring pathways linking greenspace to health: Theoretical and methodological guidance. Environ. Res..

[61] Paquet C., Coffee N.T., Haren M.T., Howard N.J., Adams R.J., Taylor A.W., Daniel M. (2014). Food environment, walkability, and public open spaces are associated with incident development of cardio-metabolic risk factors in a biomedical cohort. Health Place.

[62] Farukhi Z., Mora S. (2021). Assessing the dyslipidemias: To fast or not to fast?. Curr. Opin. Endocrinol. Diabetes Obes..

[63] Rhew I.C., Vander Stoep A., Kearney A., Smith N.L., Dunbar M.D. (2011). Validation of the normalized difference vegetation index as a measure of neighborhood greenness. Ann. Epidemiol..

[64] Browning M., Lee K. (2017). Within what distance does “Greenness” best predict physical health? A systematic review of articles with GIS buffer analyses across the lifespan. Int. J. Environ. Res. Public Health.

